# Association of *SLC11A1* polymorphisms with anthropometric and biochemical parameters describing Type 2 Diabetes Mellitus

**DOI:** 10.1038/s41598-023-33239-3

**Published:** 2023-04-16

**Authors:** Zahra Kavian, Saman Sargazi, Mahdi Majidpour, Mohammad Sarhadi, Ramin Saravani, Mansour Shahraki, Shekoufeh Mirinejad, Milad Heidari Nia, Maryam Piri

**Affiliations:** 1 Department of Nutrition, School of Medicine, Zahedan University of Medical Sciences, Zahedan, Iran; 2 Cellular and Molecular Research Center, Research Institute of Cellular and Molecular Sciences in Infectious Diseases, Zahedan University of Medical Sciences, Zahedan, Iran; 3 Clinical Immunology Research Center, Zahedan University of Medical Sciences, Zahedan, Iran; 4 Department of Clinical Biochemistry, School of Medicine, Zahedan University of Medical Sciences, Zahedan, Iran; 5 Adolescent Health Research Center, Research Institute of Cellular and Molecular Sciences in Infectious Diseases, Zahedan University of Medical Sciences, Zahedan, Iran; 6 Diabetes Center, Bu-Ali Hospital, Zahedan University of Medical Sciences, Zahedan, Iran

**Keywords:** Genetic association study, Genetic markers, Biomarkers, Metabolic disorders

## Abstract

Diabetes, a leading cause of death globally, has different types, with Type 2 Diabetes Mellitus (T2DM) being the most prevalent one. It has been established that variations in the *SLC11A1* gene impact risk of developing infectious, inflammatory, and endocrine disorders. This study is aimed to investigate the association between the *SLC11A1* gene polymorphisms (rs3731864 G/A, rs3731865 C/G, and rs17235416 + TGTG/− TGTG) and anthropometric and biochemical parameters describing T2DM. Eight hundred participants (400 in each case and control group) were genotyped using the polymerase chain reaction-restriction fragment length polymorphism (PCR–RFLP) and amplification-refractory mutation system-PCR (ARMS-PCR) methods. Lipid profile, fasting blood sugar (FBS), hemoglobin A1c level, and anthropometric indices were also recorded for each subject. Findings revealed that *SLC11A1*–rs3731864 G/A, –rs17235416 (+ TGTG/− TGTG) were associated with T2DM susceptibility, providing protection against the disease. In contrast, *SLC11A1*–rs3731865 G/C conferred an increased risk of T2DM. We also noticed a significant association between *SLC11A1*–rs3731864 G/A and triglyceride levels in patients with T2DM. In silico evaluations demonstrated that the SLC11A2 and ATP7A proteins also interact directly with the SLC11A1 protein in *Homo sapiens*. In addition, allelic substitutions for both intronic variants disrupt or create binding sites for splicing factors and serve a functional effect. Overall, our findings highlighted the role of *SLC11A1* gene variations might have positive (rs3731865 G/C) or negative (rs3731864 G/A and rs17235416 + TGTG/− TGTG) associations with a predisposition to T2DM.

## Introduction

Diabetes mellitus (DM) is a high prevailing and rapidly developing chronic endocrinological illness characterized by abnormal blood glucose levels^1^. Type 2 DM (T2DM) is a chronic disease whose global prevalence has reached worrying levels. In 2019, 463 million adults had T2DM, showing a three-fold increase worldwide compared to 20 years before the report, according to the International Diabetes Federation^2^. T2DM has a complicated etiology and is impacted by a broad spectrum of risk drivers, some of which are inevitable (such as age and genetic diversity) and others controllable (like adopting a healthy diet and exercising)^3^. Dysregulation of metabolisms of proteins, lipids, carbohydrates, and nucleic acids might cause metabolic diseases through hereditary or environmental factors. Nonalcoholic fatty liver disease (NAFLD) is also caused by excessive body fat and insulin resistance, the two most important risk factors for type 2 diabetes. Overeating, a poor diet, and a stationary lifestyle are other contributors leading to T2DM, especially in persons with genetic predispositions^4^.

The large consumption of red and processed meats, refined carbohydrates, and simple sugars defines the Western dietary pattern. This pattern has gained popularity worldwide and has been strongly related to an elevated risk of T2DM^3^. Although most of the prior investigations on T2DM were conducted in Western or European societies, it is clear that the ways the disease develops are diverse in racial groups, proposing that a one-size-fits-all perspective might not be the best when determining risk drivers. It has been acknowledged that mainstream models pursued globally, such as the Western dietary pattern and endangered environmental sustainability, raise the risk of T2DM and associated comorbidities^5^. These factors and other socioeconomic and cultural impacts are attributed to the rise in overweight, a well-known element for T2DM^6^. Genome-wide association studies (GWAS) and Mendelian randomization (MR) demonstrates that genetic polymorphisms are risk factors for human diseases^7^. Over 140 gene loci have currently been linked to T2DM by GWAS and other sequencing studies. Fifteen of these loci encode membrane transport proteins that are either known or hypothetical^8^. T2DM is a polygenic disorder that is influenced by more than 400 genetic variations, according to extensive GWAS^1,9^. There appears to be only a little predictive value in these variations over other conventional contributors, such as corpulence, a sedentary lifestyle, and poor diets in T2DM development^9–11^. Furthermore, though discrepancies in the distribution and prevalence of T2DM risk alleles have been found among races, there is little proof to support the idea that these variants account for racial dissimilarities in T2DM predisposition^1^. A growing emphasis is on how gene-environment interactions occurring during in utero development might affect the risk of developing cardio-metabolic disorders in adulthood; that is why assessing the interaction of behavior and genetics is essential. The developmental origins of the health and disease (DOHaD) framework, which has been linked to T2DM development and other non-communicable illnesses, are consistent with the current research^5,12^.

Nutritional components and environmental chemicals interact with genes to maintain regular activity in the body's complicated health system. Numerous research has been conducted on the essential nutrients and metabolites, but the mechanisms by which they are transported inside the body have received very little attention. Membrane transporters predominantly consist of ATP-binding cassettes (ABCs) and solute carrier (SLC) transporters, which are members of the ion and water channels^13^. The *Slc5*, *Slc13*, *Slc16*, *Slc25*, and *Slc30* gene families investigated in different tissues and organs, such as the pancreas, liver, gut, adrenal glands, skeletal muscle, and fat, and have been associated with metabolic diseases such as overweight, NAFLD, and T2DM in both human and ratty research^4^. It has been established that metformin's bioavailability, clearance, and pharmacological effect in T2DM are significantly influenced by the expression of the solute carrier proteins Slc22A1, Slc22A2, Slc22A3, and Slc47A1^14,15^. There has yet been no mention of a thorough analysis of *SLC* genes in obesity^16^.

In humans, the host resistance factor *SLC11A1* [formerly known as natural resistance-associated macrophage protein 1 (NRAMP1)] is abundantly expressed in monocytes and phagocytes^17^. The gene encoding *SLC11A1* in humans is 14 kb in size and contains 15 exons. This gene clusters with other genes in close proximity to its location on chromosome 2q35, in a region of high linkage disequilibrium (LD) that spans about 400 kb^18^. SLC11A1 has been shown to control the susceptibility to *Salmonella*, *Mycobacterium*, and *Leishmania* infections within cells^19^. SLC11A1's role in the discharge of Fe^2+^, Mn^2+^, and Co^2+^ from phagosomes may prevent vacuolar pathogens' access to these essential micronutrients^20^. According to a study by Yang et al. the *SLC11A1*–rs3731685 G/A variation might correlate with Type 1 DM (T1DM) risk in a large-scale survey of 8463 cases and 9835 controls^18^.

**Figure 1:**
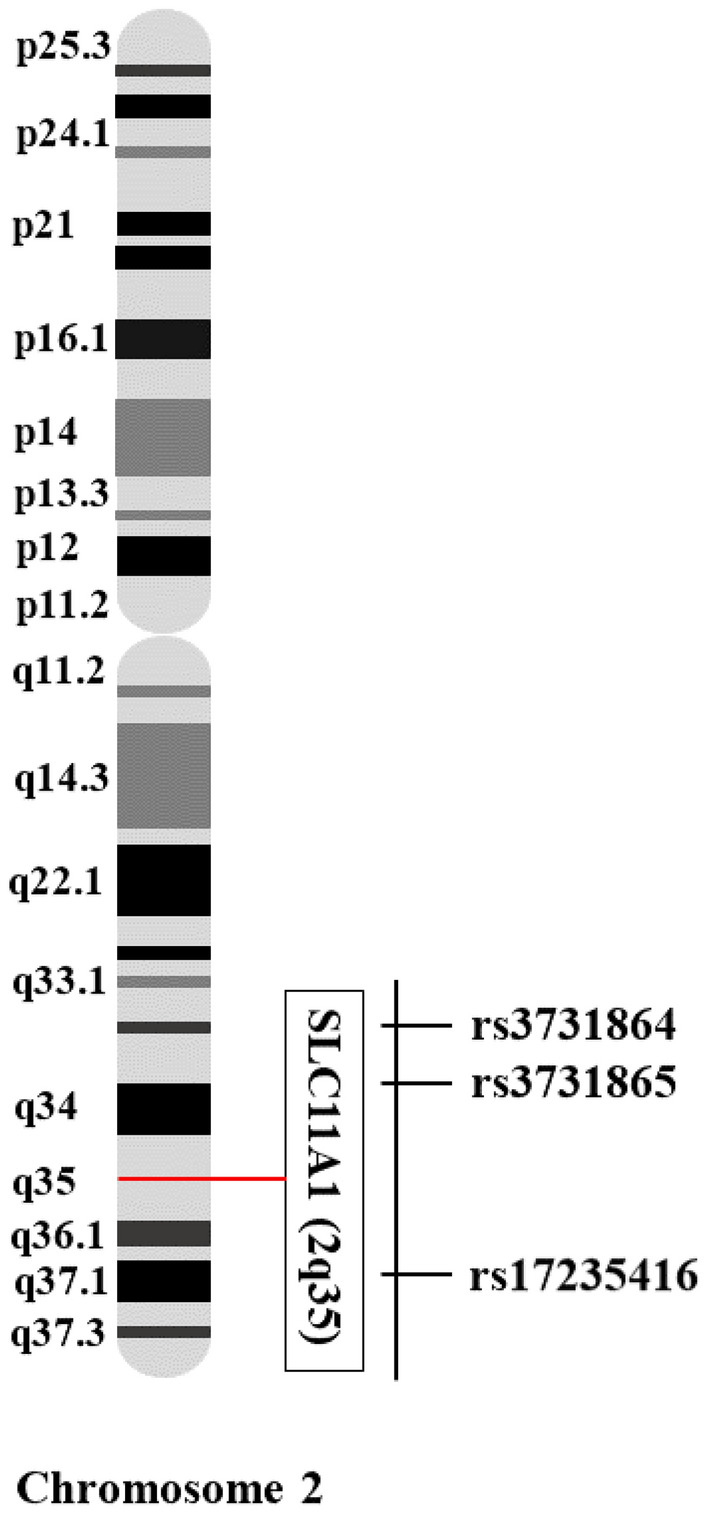
Loci of the studied *SLC11A1* gene polymorphisms on chromosome 2 (2q35).

One of the *SLC11A1* variations known to alter *SLC11A1* transcription and functioning is the INT4 polymorphism (469 + 14G/C or rs3731865), which is positioned in exon 4a^21,22^. Furthermore, the 3'UTR (1729 + 55del4 or rs17235416) variation refers to a 4 bp insertion/deletion immediately 3' of the stop codon^23,24^; nevertheless, the potential role of this polymorphism on the functioning and expression of *SLC11A1* has not yet been established. With this background, the present study aimed to unveil the possible correlation between *SLC11A1* variants, including rs3731865G/C, rs3731864 (577-18G/A) in intron 5, and rs17235416 + TGTG/−TGTG polymorphisms and the risk of T2DM in an Iranian population. Figure 1 schematically represents the location of the studied variations on chromosome 2.

## Methods

### Participants and study protocols

Eight hundred participants (400 in each case and control group) were selected among individuals referred to the Diabetes Clinic of Bu-Ali Hospital, Zahedan, Iran. The case group was of individuals with T2DM who had fasting blood sugar (FBS) levels of ≥ 126 mg/dL and hemoglobin A1c (HbA1c) levels of ≥ 6.5%^25^. The diagnosis was made according to the 2018 American Diabetes Association (ADA) Standards of Medical Care in Diabetes. The healthy, non-diabetic control group was selected from those with FBS levels < 99 mg/dL and HbA1c levels < 5.7% (to weed out pre-diabetic ones). Individuals with late-stage autoimmune or malignant diseases, gestational diabetes, polycystic ovary syndrome, metabolic syndrome, chronic renal failure, hypertension, and pregnant women were excluded from the study. The human-involved procedure was under the 1964 Helsinki declaration, and the ethics committee of Zahedan University of Medical Sciences approved the study's protocols (ethical code: IR.ZAUMS.REC.1400.028). The webpage of the ethics certificate is available at https://ethics.research.ac.ir/EthicsProposalViewEn.php?id=189533. Before enrollment, informed consent was obtained from all subjects or their legal guardians.

### Sample collection, sample size, biochemical assessments, and anthropometric parameters

For each participant, a total of 5 mL of whole blood was drained in ethylenediaminetetraacetic acid (EDTA)-containing- or serum clot activator tubes. The former tube was used for DNA extraction and HbA1c measurement, and the latter was utilized for measuring FBS and lipid indices [including triglyceride (TG), total cholesterol (TC), high-density lipoprotein (HDL-c), low-density lipoprotein (LDL-c)] using commercial spectrophotometric kits (PishtazTeb Diagnostics®, Tehran, Iran).

To calculate sample size, we conducted a pilot study to collect blood samples from a small population (100 participants, including 50 T2DM patients and 50 healthy subjects) and genotyped all of the examined SNPs. This allowed us to identify an adequate sample size. The chi-square test was then used to calculate the allelic frequencies of the investigated variants in both groups. The estimated frequencies were then subjected to a sample size analysis utilizing the sample size calculator server's online version (available at: https://clincalc.com/stats/SampleSize.aspx). The server uses the below formula to calculate sample size.

$$n=\frac{{\left({z}_{1- \frac{\alpha }{2}} + {z}_{1- \beta }\right)}^{2}\left({P}_{1}\left(1-{P}_{1}\right)+ {P}_{2}\left(1-{P}_{2}\right)\right)}{{\left({P}_{1}- {P}_{2} \right)}^{2}}$$

where P1 represents the frequency of the wild or mutant allele in control, P2 is the frequency of the wild or mutant allele in case, Z is the critical Z value for a given α or β, α indicates the probability of type I error (usually 0.05), and β is considered the probability of type II error (usually 0.2). The calculator was used to determine the sample size for the tested variations in the studied groups, with study power set to 80%. The threshold of sample size was then adjusted for a total of 800 subjects.

The weight and height were measured twice for each person to calculate the BMI, and the average was considered for the final measure. For this, weight was determined with minimum clothing, without shoes, and with a standard weight gauge with an accuracy of 100 g. A standard meter with an accuracy of 0.1 cm was also utilized to calculate the height while the person was barefoot and placed next to the behind-the-leg gauge. Moreover, the narrowest waist area between the lowermost rib and the iliac crest above the navel was metered as waist circumference (WC) with an inelastic measuring tape to within 0.1 cm. The widest hip area and its maximum bulge were determined to measure the hip circumference with an accuracy of 0.1 cm. Waist-to-hip ratio (WHR) was calculated by dividing the waist circumference by the hip circumference in centimeters. The conicity index (CI) was calculated as previously described by Shidfar et al.^26^. Table 1 summarizes the clinical and demographic characteristics of all participants.

**Table 1:** Clinical and demographic data of patients with T2DM and healthy controls.

Parameter	T2DM, n (mean ± SD)	Control, n (mean ± SD)	*p-*value
Gender			0.819^b^
Male	126	123	
Female	274	277	
Age (year)	54.4 ± 9.7	53.4 ± 9.6	0.058^a^
BMI (kg/m^*2*^*)*	26.2 ± 2.8	23 ± 1.4	**< 0.001**^**a**^
Underweight or < 18.5	2	1	**–**
Ideal or 18.5–24.9	127	363	**–**
Overweight or 25.0–29.9	255	36	**–**
Obese or ≥ 30	16	0	**–**
WC (cm)	1.01 ± 0.11	0.84 ± 0.09	**< 0.001**^**a**^
WHR	0.91 ± 0.03	0.93 ± 0.27	0.439^a^
CI (m^*2*^/kg)	1.41 ± 0.06	1.26 ± 0.22	**< 0.001**^**a**^
FBS	168.6 ± 56.84	94.09 ± 8.26	**< 0.001**^**a**^
TC	202.24 ± 66.78	150.49 ± 37.71	**< 0.001**^**a**^
TG	162.48 ± 83.26	119.62 ± 64	**< 0.001**^**a**^
HDL	56.67 ± 21.89	48.66 ± 14.01	**< 0.001**^**a**^
LDL	118.78 ± 42.35	93.88 ± 25.90	**< 0.001**^**a**^

*T2DM* Type 2 Diabetes Mellitus, *n* represents the numbers, *BMI* body mass index, *WC* waist circumference, *WHR* waist-to-hip ratio, *CI* conicity index, *FBS* fasting blood sugar, *TC* total cholesterol, *TG* triglyceride, *HDL* high-density lipoprotein, *LDL* low-density lipoprotein, ^a^Mann–Whitney-Wilcoxon test; ^b^Pearson Chi-Square test. Statistically significant parameters (*p*-value < 0.05) are shown in Bold and Italics.

### Genomic DNA isolation and genotyping

Genomic DNA was extracted from nucleated white blood cells using a simple salting-out technique^27^. The purity and concentration of extracted DNA were determined by calculating the 260/280 optical density ratio using a Nanodrop device (Maestrogen®, Taiwan). Data for selecting variations and designing specific primers were acquired from National Center for Biotechnology Information (*NCBI*) database. Specific primers were designed using the Gene Runner® v.6.5.52 Beta software and produced by GenFanAvaran Company in Iran.

Studied variations were genotyped by applying the polymerase chain reaction-restriction fragment length polymorphism (PCR–RFLP) (for *SLC11A1*–rs3731864 and –rs3731865 SNPs) and amplification-refractory mutation system-PCR (ARMS-PCR) (for *SLC11A1*–rs17235416) techniques. The reaction mixture had a final volume of 20 μL and contained 0.9 μL of genomic DNA (~ 60 ng/mL), 0.8 μL of each primer (8 pmol), 10 μL of 2 × Taq PreMix (Parstous Biotechnology®, Mashhad, Iran), and 7.5 μL of double-distilled water. The mixture was cycled using a Techne thermal cycler (Techne, US) under the following conditions: initial denaturation at 95 °C for 5 min, 35 cycles at 94 °C for 30 s, specific annealing temperatures (based on Supplementary Table S1 for each variation) for 30 s, and an extension step at 72 °C for 30 s. These stages were followed by a final extension step at 72 °C for 5 min.

**Figure 2:**
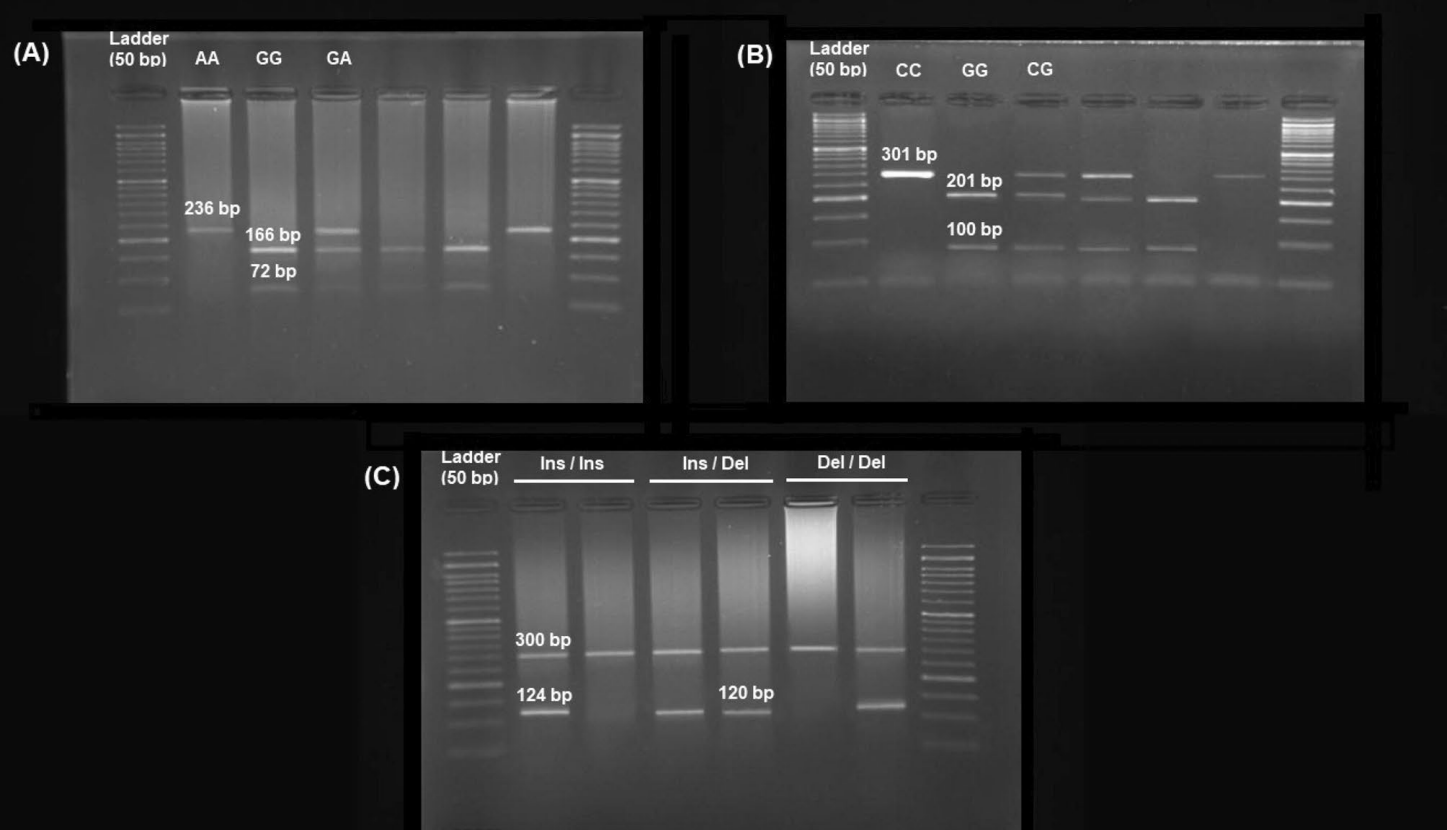
Gel photograph of *SLC11A1*–rs3731864 G/A (**A**), –rs3731865 G/C (**B**), and –rs17235416 + TGTG/− TGTG (**C**) polymorphisms.

The PCR product was subjected to *MspI* (for *SLC11A1*–rs3731864 G/A) or *ApaI* (for *SLC11A1*–rs3731865 G/C) restriction enzymes (ThermoFisher®, Massachusetts, U.S.A.) and incubated for 10 h at 37 °C. PCR products were then electrophoresed on 1.5% agarose gel stained by GreenViewer dye (Parstous, Mashhad, Iran). DNA bands were photographed under ultraviolet (Fig. 2). Random genotyping was performed on 30% of the samples, and genotyping accuracy was found to be > 99%.

### Statistical analyses

SPSS version 22.0 software (SPSS, Inc., Chicago, IL, USA) was recruited for data analysis. Deviation from Hardy–Weinberg Equilibrium (HWE) was assessed via Pearson's Chi-square test. Continuous variables were compared between cases and controls using standard single sample t statistic, Mann–Whitney–Wilcoxon, and Pearson Chi-Square tests where appropriate and expressed as mean ± standard deviation (SD). Odds ratio (OR) and 95% confidence intervals were calculated to estimate the relative risk of the disease. Binary logistic regression analysis was employed to examine the correlation between the clinical-demographic findings of the studied groups and T2DM risk. Besides, haplotype analysis was conducted through the online SHEsis software^28^. A *p*-value less than 0.05 was considered statistically significant.

### Computational analyses

A complex set of RNA-binding proteins controls the post-transcriptional processing of RNA, such as capping, polyadenylation, splicing, export, and the protein's secondary structure. Allelic substitution in DNA can affect some of these processes, primarily the accuracy and efficiency of splicing, by altering the complex of proteins bound to the pre-mRNAs. Knowing the RNA sequences recognized by each protein involved in post-transcriptional RNA processing is necessary for predicting the effects of mutations at both RNA and protein levels. For this purpose, we recruited the SpliceAid database to determine the impact of studying both intronic variants of *SLC11A1* on the pattern of splicing processes^29^. SpliceAid is a web-based tool collecting all the experimentally assessed target RNA sequences that are bound by splicing proteins in humans. Using the SpliceAid server, the user submits sequences, and the server identifies the exact correspondence between the sequences submitted and the sequences in the database, giving accurate and dynamic graphic results.

The *WebLogo* v.2.8.2 server was employed to identify the preserved regions of all three studied polymorphisms^30^. Using WebLogo, sequence logos are generated, representing patterns within multiple sequence alignments. In comparison with consensus sequences, sequence logos provide a more detailed and more accurate description of sequence similarity and can be used to quickly reveal characteristics of an alignment that would otherwise be difficult to detect. An individual logo consists of a stack of letters at each position in the sequence, one stack for each letter. Stack heights (measured in bits) represent sequence conservation at each position, and symbol heights reflect the relative frequency of amino acids or nucleic acids at each position^30^. As defined by Schneider and Stephens, sequence conservation is the difference between the entropy of the observed symbol distribution and the maximum possible entropy^31^. In particular, sequence logos offer a richer and more detailed description of, for example, a binding site, as compared with consensus sequences^32^. Sequence information of genomic DNA in different formats, including CLUSTALW, FASTA, MSF, NBRF, PIR, NEXUS, PHYLIP, and plain flat-file, can be entered into the Weblogo server (available at https://weblogo.threeplusone.com/create.cgi) for multiple sequence alignments. Depending on how frequently they occur, various SNPs are scaled.

To predict the protein–protein interaction (PPI) network of the SLC11A1 protein, the newest version (01-04-2022) of the web-based inBio Discover™ database (https://inbio-discover.intomics.com/map.html) was employed^33^. The database is a comprehensive and accurate PPI resource built from more than six million traceable entries, showing a set of highly trusted interactions between proteins based on experimentally determined databases. This research put the network expansion in the “Include neighboring proteins” mode to show close and related proteins in terms of expression, regulation, or function. In this connection, pathway interactions are indicated as lines, and the remainder is inBio Map™ high-confidence interactions. Data were generated by entering the UniProt ID for the SLC11A1 protein, P49279, into the server. UniProt is an online reservoir for proteins that extracts data from the Swiss-Prot, TrEMBL, and PIR-PSD databases^33,34^. Expression, regulatory, and function-related proteins were made available through the network expansion method of this database. In order to design an interaction network for *SLC11A1* as a hub gene, information regarding the known and/or predicted interactions, gene fusion, co-expression, and protein homology was obtained using STRING^34^. STRING imports protein association knowledge from databases of physical interactions and databases of curated biological pathway knowledge (MINT, HPRD, BIND, DIP, BioGRID, KEGG, Reactome, IntAct, EcoCyc, NCI-Nature Pathway Interaction Database, and GO). Finally, inBio Discover™ was utilized to analyze the PPI network.

## Results

### Laboratory and demographic findings

The case group consists of 400 patients with T2DM (274 women and 126 men; mean age of 54.4 ± 9.7) and 400 healthy control subjects (277 females and 123 males; the average age of 53.4 ± 9.6). As shown in Table 1, no marked difference was noticed between the studied groups concerning age, gender, and WHR (*p* = 0.058, 0.819, and 0.439, respectively). At the same time, FBS, TG, TC, HDL-c, LDL-c, conicity index, and body mass index (BMI) were significantly different between cases and controls (*p* < 0.001).

### Genetic association analysis

Table 2 shows the genotypic and allelic distribution of the studied *SLC11A1* gene variants in controls and T2DM cases. None of the studied variations deviated from HWE in cases or controls (*p-value* for HWE > 0.05). We found a strong link between the rs3731864 G/A variant and T2DM under codominant1 GA vs. GG (OR 0.43; 95% CI 0.28–0.66; *p* < 0.001), dominant GA + AA vs. GG (OR 0.43; 95% CI 0.28–0.65; *p* < 0.001), and overdominant GA vs. GG + AA (OR 0.43; 95% CI 0.28–0.67; *p* < 0.001) genetic patterns. Moreover, the A allele of rs3731864 G/A decreased T2DM risk by 54%. Similarly, The rs17235416 variant was associated with a decrease in T2DM risk under codominant1 Ins/Del vs. Ins/Ins (OR 0.48; 95% CI 0.27–0.83; *p* < 0.009), dominant Ins/Del + Del /Del vs. Ins/Ins (OR 0.47; 95% CI 0.27–0.80; *p* < 0.006), and over-dominant Ins/Del vs. Ins/Ins + Del /Del (OR 0.48; 95% CI 0.28–0.84; *p* < 0.010) modes of inheritance. Deletion of the TGTG repeat in this polymorphism conferred protection against T2DM (OR 0.47; 95% CI 0.28–0.79; *p* < 0.004). In contrast, compared with the healthy controls, T2DM risk was dramatically increased in patients carrying the CG (OR 1.53; 95% CI 1.07–2.19; *p* < 0.019), CG + GG (OR 1.52; 95% CI 1.07–2.16; *p* < 0.020) genotypes of rs3731865 G/C. Furthermore, an increase in T2DM risk was found under the allelic (G vs. C) as well as the overdominant (CG vs. CC + GG) model of this single nucleotide variation (SNP) [OR 1.44; 95% CI 1.03–2.00; p < 0.029 and OR 1.53; 95% CI 1.07–2.19; p < 0.019, respectively].

**Table 2:** Allelic and genotypic distribution of *SLC11A1* gene polymorphisms.

Polymorphism	T2DM, n (%)	Control, n (%)	Genetic model	OR (95% CI)*	*p* value*
rs3731864 G/A					
GG	360 (90.0)	321 (80.3)		1 [reference]	–
GA	37 (9.2)	73 (18.2)	Codominant 1	0.43 (0.28–0.66)	**< 0.001**
AA	3 (0.8)	6 (1.5)	Codominant 2	0.46 (0.11–1.86)	0.278
HWE	0.095	0.434	Dominant	0.43 (0.28–0.65)	**< 0.001**
			Recessive	0.52 (0.13–2.08)	0.353
			Over Dominant	0.43 (0.28–0.67)	**< 0.001**
G	757 (94.6)	715 (89.4)	Allelic	1 [reference]	–
A	43 (5.4)	85 (10.6)	Allelic	0.46 (0.31–0.68)	**< 0.001**
rs3731865 C/G					
CC	303 (75.8)	333 (83.3)		1 [reference]	–
CG	95 (23.7)	65 (16.2)	Codominant 1	1.53 (1.07–2.19)	**0.019**
GG	2 (0.5)	2 (0.5)	Codominant 2	1.13 (0.16–8.09)	0.901
HWE	0.064	0.75	Dominant	1.52 (1.07–2.16)	**0.020**
			Recessive	1.04 (0.15–7.43)	0.967
			Over Dominant	1.53 (1.07–2.19)	**0.019**
C	701 (87.6)	731 (91.4)	Allelic	1 [reference]	–
G	99 (12.4)	69 (8.6)	Allelic	1.44 (1.03–2.00)	**0.029**
rs17235416 + TGTG/−TGTG					
+ TGTG/+ TGTG	378 (94.5)	356 (89.0)		1 [reference]	
+ TGTG/− TGTG	21 (5.2)	41 (10.2)	Codominant 1	0.48 (0.27–0.83)	**0.009**
− TGTG/− TGTG	1 (0.3)	3 (0.8)	Codominant 2	0.33 (0.03–3.16)	0.334
HWE	0.28	0.15	Dominant	0.47 (0.27–0.80)	**0.006**
			Recessive	0.35 (0.04–3.34)	0.358
			Over Dominant	0.48 (0.28–0.84)	**0.010**
+ TGTG	777 (97.1)	753 (94.1)	Allelic	1 [reference]	**–**
− TGTG	23 (2.9)	47 (5.9)	Allelic	0.47 (0.28–0.79)	**0.004**

*T2DM* Type 2 Diabetes Mellitus, *n* represents the numbers, *CI* confidence intervals, *OR* odds ratio, *HWE* Hardy–Weinberg equilibrium, *BMI* body mass index, *p-value and OR (95% CI), BMI-adjusted. Codominant 1 and Codominant 2 represent the heterozygous and homozygous codominant models, respectively. *p* < 0.05 were considered statistically significant, indicated in Bold.

The correlation between the *SLC11A1* SNPs and laboratory findings and the demographical characteristics of the studied groups are shown in Table 3. We noticed a significant association between *SLC11A1*–rs3731864 G/A and TG levels in patients with T2DM (*p* = 0.048). The *SLC11A1*–rs3731865 C/G variant was associated with WC and LDL-c levels of the healthy controls (*p* = 0.046 and 0.006, respectively). Moreover, the *SLC11A1*–rs17235416 Ins /Del variant was associated with WC, conicity index, and HDL-c levels in controls (*p* = 0.018, 0.027, and 0.025, respectively).

**Table 3:** Association between *SLC11A1* variants and clinical-demographic characteristics of patients with T2DM and healthy subjects.

SNP	Group	Genotype	BMI	WC	WHR	CI	FBS	TG	TC	HDL-c	LDL-c
rs3731864 G/A	T2DM	GG + GA	26.25 ± 2.83	1.01 ± 0.11	0.91 ± 0.031	1.41 ± 0.06	168.5 ± 57.02	164.5 ± 86.6	202.3 ± 66.91	56.77 ± 21.91	119.0 ± 42.42
		AA	25.72 ± 3.37	1.03 ± 0.15	0.92 ± 0.004	1.43 ± 0.05	181.6 ± 25.79	965 ± 13.52	194.3 ± 55.90	44.46 ± 19.53	91.33 ± 23.03
		***p*-value**	0.750	0.804	0.427	0.457	0.690	**0.048**	0.837	0.333	0.260
	Control	GG + GA	23.04 ± 1.38	0.84 ± 0.09	0.93 ± 0.27	1.26 ± 0.22	94.07 ± 8.24	120.1 ± 64.1	150.74 ± 37.7	48.68 ± 14.04	94.01 ± 25.81
		AA	22.33 ± 1.47	0.88 ± 0.07	1.01 ± 0.22	1.35 ± 0.18	95.16 ± 10.45	885.6 ± 39.75	134.16 ± 32.67	47.33 ± 12.11	85.33 ± 33.20
		***p*-value**	0.212	0.344	0.465	0.332	0.749	0.160	0.286	0.815	0.416
rs3731865 C/G	T2DM	CC + CG	26.23 ± 2.83	1.01 ± 0.11	0.91 ± 0.030	1.41 ± 0.07	168.6 ± 56.98	163.5 ± 86.8	202.2 ± 66.92	56.7 ± 21.93	118.9 ± 42.30
		GG	29.52 ± 0.64	1.11 ± 0.04	0.90 ± 0.003	1.41 ± 0.02	160.0 ± 5.65	194.0 ± 115.9	210 ± 33.94	45.0 ± 7.07	96.5 ± 68.59
		***p*-value**	0.101	0.236	0.606	0.900	0.830	0.622	0.869	0.450	0.456
	Control	CC + CG	23.02 ± 1.38	0.84 ± 0.09	0.93 ± 0.27	1.26 ± 0.22	94.11 ± 8.27	119.6 ± 64.07	150.4 ± 37.78	48.65 ± 14.04	94.13 ± 25.71
		GG	24.74 ± 0.95	0.70 ± 0.03	0.62 ± 0.05	0.96 ± 0.07	89.00 ± 7.07	111.5 ± 62.9	167 ± 11.31	50.5 ± 0.70	43.5 ± 9.19
		***p*-value**	0.080	**0.046**	0.108	0.056	0.383	0.885	0.536	0.853	**0.006**
rs17235416 Ins/Del	T2DM	ins/ins + ins/del	26.24 ± 2.84	1.01 ± 0.11	0.91 ± 0.03	1.41 ± 0.07	168.6 ± 56.91	163.6 ± 86.90	202.2 ± 66.85	56.7 ± 21.92	118.7 ± 42.37
		del/del	26.76 ± 0.0	1.03 ± 0.0	0.93 ± 0.0	1.42 ± 0.0	153 ± 0.0	191 ± 0.0	232 ± 0.0	47 ± 0.0	154 ± 0.0
		***p*-value**	0.854	0.886	0.656	0.816	0.784	0.754	0.656	0.659	0.406
	Control	ins/ins + ins/del	23.02 ± 1.38	0.84 ± 0.09	0.93 ± 0.27	1.26 ± 0.22	94.06 ± 8.24	119.9 ± 64.1	150.36 ± 37.79	48.59 ± 14.03	93.95 ± 25.96
		del/del	24.44 ± 1.29	0.71 ± 0.07	0.63 ± 0.12	0.98 ± 0.14	97.33 ± 13.31	482 ± 2.64	168.33 ± 21.07	57 ± 3	84.33 ± 15.37
		***p*-value**	0.077	**0.018**	0.056	**0.027**	0.174	0.534	0.412	**0.025**	0.522

*T2DM* Type 2 Diabetes Mellitus, *BMI* body mass index, *WC* waist circumference, *WHR* waist-to-hip ratio, *CI* conicity index, *FBS* fasting blood sugar, *TC* total cholesterol, *TG* triglyceride, *HDL-c* high-density lipoprotein c, *LDL-c* low-density lipoprotein c, *Ins* insertion, *Del* deletion. *p*-values < 0.05 were considered statistically significant and shown in Bold.

### Haplotype and interaction analysis

Supplementary Table S2 represents the association between *SLC11A1*–rs3731864 G/A, –rs3731865 G/C, and –rs17235416 +TGTG/−TGTG haplotypes in T2DM cases and healthy controls. We found a higher frequency of the *SLC11A1*–rs3731864 G/A, –rs3731865 G/C, and –rs17235416 +TGTG/−TGTG haplotypes in patients with T2DM compared with controls. Compared with the reference haplotype (G/C/ + TGTG), the A/C/ + TGTG haplotype of rs3731864/rs373186/rs17235416 significantly diminished T2DM risk in our population (OR 0.84, 95% CI 0.27–0.85, and *p* = 0.043). The linkage disequilibrium (LD) between three *SLC11A1* polymorphisms was also calculated, and no strong LD was found between the three studied variations (Supplementary Fig. S1).

Table 4 summarizes the interaction analysis of *SLC11A1* polymorphisms on T2DM risk. Compared with the reference combination (GG/GC/Ins-Del), the genotype combination of GA/CC/Ins-Ins markedly increased T2DM risk by 1.66 folds (OR 1.66, CI 1.11–2.48, and *p* = 0.013), whereas the GA/CC/ Ins-Ins combination diminished T2DM risk by 57% (OR 0.43, 95% CI 0.26–0.71, and *p* < 0.001).

**Table 4:** Interaction analysis of *SLC11A1* polymorphisms on T2DM risk.

rs3731864 G/A	rs3731865 C/G	rs17235416 + TGTG/− TGTG	Case (%)	Control (%)	OR (95% CI)	*p*-value
GG	CC	+ TGTG/+ TGTG	255 (63.8)	235 (58.8)	1 [reference]	
GA	CC	+ TGTG/+ TGTG	26 (6.5)	55 (13.8)	0.43 (0.26–0.71)	** < 0.001**
GG	CG	+ TGTG/+ TGTG	83 (20.8)	46 (11.5)	1.66 (1.11–2.48)	**0.013**
GG	CC	+ TGTG/− TGTG	17 (4.3)	31 (7.8)	0.50 (0.27–0.94)	0.028
GA	CG	+ TGTG/+ TGTG	9 (2.3)	12 (3.0)	0.70 (0.29–1.67)	0.410
AA	CC	+ TGTG/+ TGTG	3 (0.8)	5 (1.3)	0.55 (0.13–2.34)	0.415
GA	CC	+ TGTG/− TGTG	2 (0.5)	5 (1.3)	0.37 (0.07–1.92)	0.218
GG	CG	+ TGTG/− TGTG	2 (0.5)	5 (1.3)	0.37 (0.07–1.92)	0.218
GG	GG	+ TGTG/+ TGTG	2 (0.5)	2 (0.5)	0.92 (0.13–6.60)	0.935
GG	CG	− TGTG/− TGTG	1 (0.3)	1 (0.3)	0.92 (0.05–14.82)	0.954

*T2DM* Type 2 Diabetes Mellitus, *OR* odds ratio, *CI* confidence intervals. *p* < 0.016 was considered statistically significant, shown in Bold.

### Computational predictions

**Figure 3:**
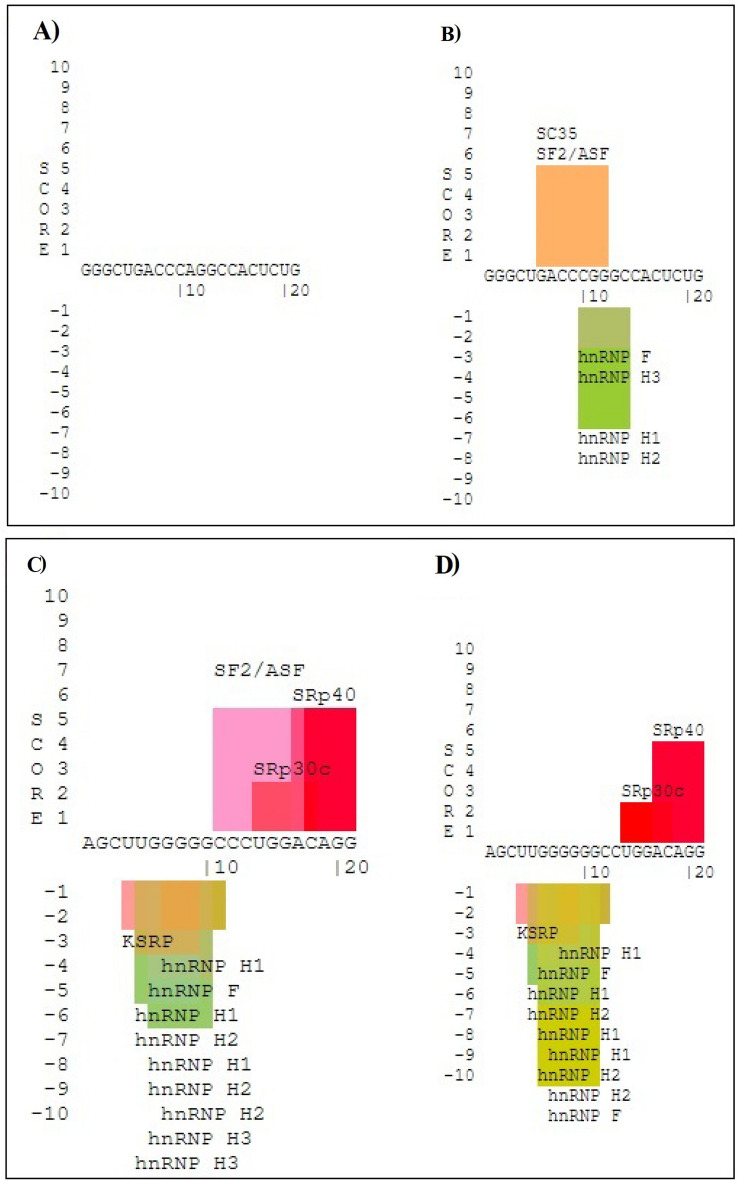
Web-based analysis of the impact of studied intronic variants on the pattern of splicing processes using the SpliceAid database. rs3731864 mutant (**A**), rs3731864 wild-type (**B**), rs3731865 mutant (**C**), rs3731865 wild-type (**D**). G to A substitution in the rs3731864 position disrupts the binding sites of some splicing factors including SC35, SF2/ASF, hnRNP f, hnRNP H3, hnRNP H1, and hnRNP H2. On the contrary, nucleotid change on the position of rs3731865 creates binding site for SF2/ASF and hnRNP H3 facors.

**Figure 4:**
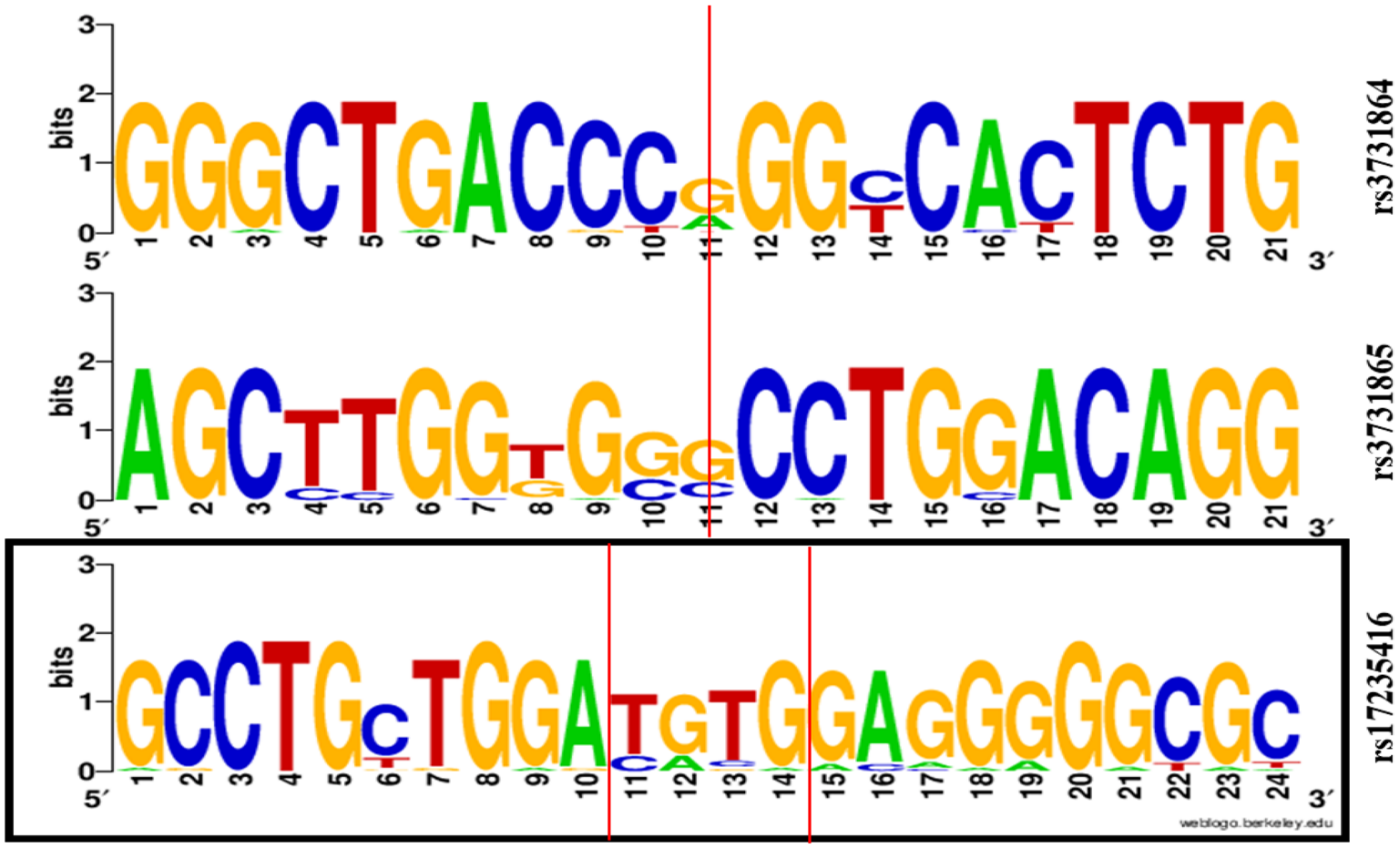
Illustration of sequence conservation. *WebLogo* illustrated the conservation of the DNA sequences around *SLC11A1*–rs3731864 G/A, –rs3731865 G/C, and –rs17235416 + TGTG/− TGTG variations loci. The red vertical line indicates the positions of the variants loci in humans and the conservation of wild alleles across multiple mammalian species. The high nucleotide symbols indicate more conservation, while the small and more diverse ones show less conservation.

**Figure 5:**
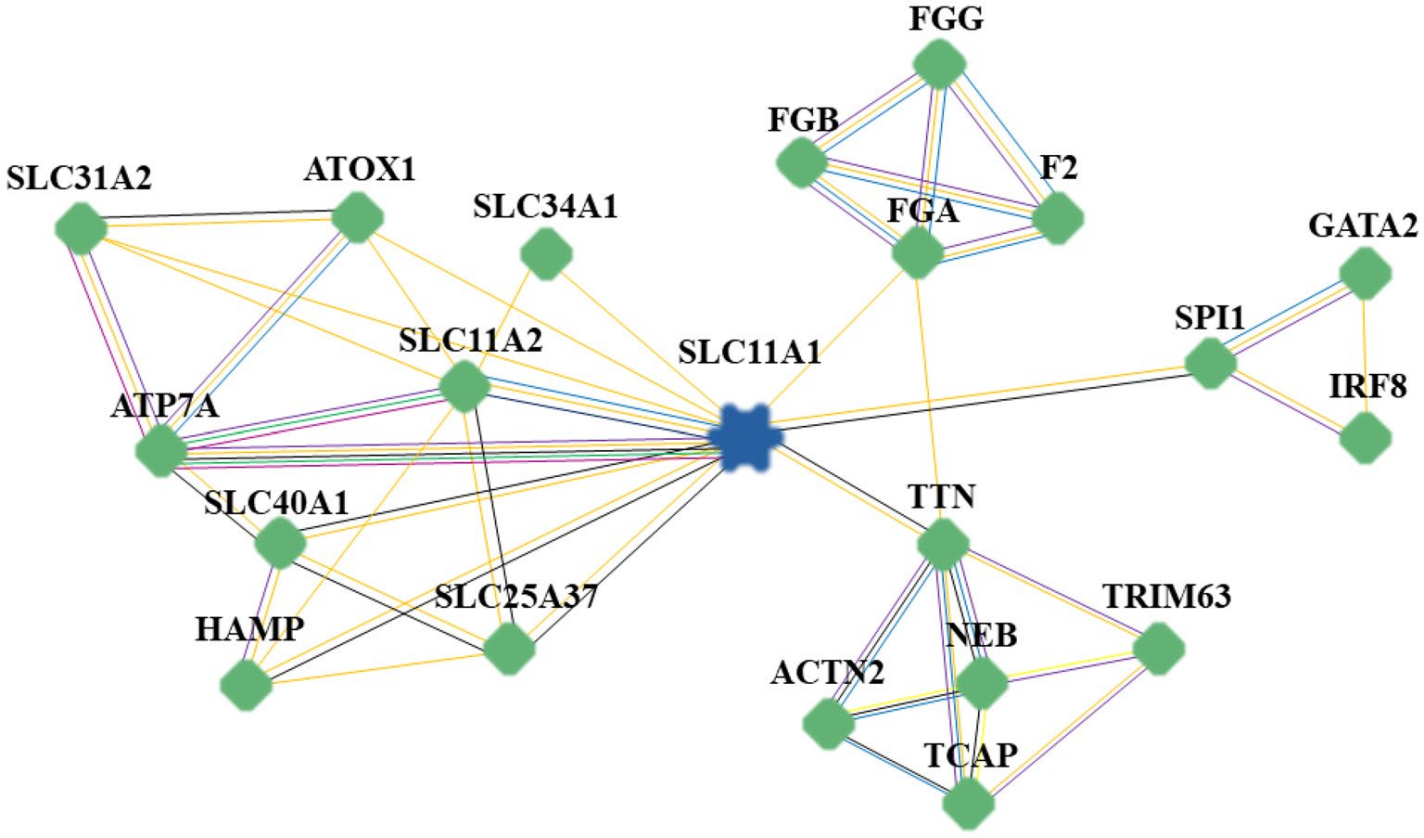
The PPI network of the SLC11A1 protein. Colored lines between proteins indicate evidence of various types of interactions. Regarding “Known Interactions”, the blue lines represents interactions based on curated databases and the purple lines highlight interactions based on experimentally determined. Regarding “Predicted Interactions”, the green lines show interactions based on gene neighborhood; the red lines indicate gene fusions; and the dark blue, represents gene co-occurrence. Regarding “Others,” the yellow lines represents interaction based on text mining; the black lines show co-expression; and the light blue lines represent protein homology. The above classification information was obtained using STRING. STRING imports protein association knowledge from databases of physical interactions and databases of curated biological pathway knowledge (*MINT*, *HPRD*, *BIND*, *DIP*, *BioGRID*, *KEGG*, *Reactome*, *IntAct*, *EcoCyc*, *NCI-Nature Pathway Interaction Database*, and *GO*). A PPI analysis was conducted using *inBio Discover™* to investigate the possible interactions between SLC11A1 and other proteins. The PPI analysis was conducted using inBio Discover™ to investigate possible interactions between SLC11A1 and other proteins. Our Bioinformatics results showed that some of the genes that have direct interaction with SLC11A1, including ATP7A, FGB, FGA, SLC11A2, and SLC40A1, can also play important roles in the course of T2DM, and this makes SLC11A1 a hub protein that can regulate different signaling pathways involved in the pathogenesis of T2DM. *PPI* protein–protein interaction, *SLC11A1* solute carrier family 11 member 1, *SLC11A2* solute carrier family 11 member 2, *SLC25A37* solute carrier family 25 member 37, *SLC31A2* solute carrier family 31 member 2, *SLC34A1* solute carrier family 34 member 1, *SLC34A2*, solute carrier family 34 member 2, *SLC40A1* solute carrier family 40 member 1, *ATOX1* antioxidant 1 copper chaperone, *ATP7A* ATPase copper transporting alpha, *HAMP* hepcidin antimicrobial peptide, *FGA* fibrinogen alpha chain, *FGB* fibrinogen beta chain, *FGG* fibrinogen gamma chain, *F2* coagulation factor II, thrombin, *SPL1* squamosa promoter binding protein-like 1, *IRF8* interferon regulatory factor 8, *GATA2* GATA binding protein 2, *TTN* titin, *NEB* nebulin, *TRIM63* tripartite motif containing 63, *ACTN2* actinin alpha 2, *TCAP* titin-cap.

The results of the SpliceAid server showed that G to A substitution in the rs3731864 position disrupts the binding sites of some splicing factors, including SC35, SF2/ASF, hnRNP F, hnRNP H3, hnRNP H1, and hnRNP H2. On the contrary, nucleotide change on the position of rs3731865 creates a binding site for SF2/ASF and hnRNP H3 factors (Fig. 3). Variation analysis using the *WebLogo* server demonstrated that all three studied polymorphisms, especially *SLC11A1*–rs3731864 G/A and –rs3731865 G/C, resided in unconserved regions across multiple mammalian species (Fig. 4). Furthermore, the *inBio Discover™* databank revealed that solute carrier family 11 member 2 (SLC11A2) and ATPase copper transporting alpha (ATP7A) proteins have direct interactions with the SLC11A1 protein in *Homo Sapiens*. According to the known interactions (from curated databases and experimentally ascertained), the ATP7A in and of itself interacts with SLC11A2, solute carrier family 31 member 2 (SLC31A2), and antioxidant 1 copper chaperone (ATOX1) in *Homo sapiens* (Fig. 5).

## Discussion

In the last 30 years, the prevalence of T2DM has doubled, making it one of the most significant global health issues^35^, suggesting the urgent need to identify novel biomarkers for early diagnosis of this endocrine disease. Genetic variations located in the intronic region^36^ or the 3′-Untranslated Region (UTR)^37,38^ of some genes have been found to independently contribute to the predisposition to T2DM, suggesting that there may be other, as-yet-undiscoverable functional variations in *Homo sapiens*. Additional in-depth population genetic research will be required to comprehend the connection between more complicated haplotypes and disease development in various geographical areas^39^. In the present work, for the first time, we sought to investigate the correlation between *SLC11A1* variants and the risk of T2DM in a sample of the Iranian population. Our findings showed a significant association between *SLC11A1* polymorphisms and T2DM risk, where rs3731865 G/C markedly enhanced T2DM risk and both rs3731864 G/A and rs17235416 + TGTG/− TGTG variants significantly diminished the risk of this endocrine diseases under different genetic models.

In terms of *SLC11A1*, most previous reports focused on the role of *SLC11A1* variants in the pathogenesis of autoimmune and infectious diseases^40^, such as HIV^41^. It was also suggested that the (GT)n polymorphism's high-expressing allele (iii) and low-expressing allele (ii), respectively, might be responsible for susceptibility to these conditions. This has also been confirmed in numerous studies on autoimmune and infectious diseases, including tuberculosis, demonstrating that there may be some balance in selecting factors that maintain both alleles in the population^39^. In another study by Ling et al. (2014), it was reported that the A allele in *SLC6A20*–rs13062383 increases the susceptibility to T2DM in populations with different genetic backgrounds^42^. Xu et al. concluded that T2DM is associated with the AA genotype of *SLC30A8*–rs11558471 in *Homo sapiens*. Haplotype A/C/A seems to be a risk factor, and haplotype A/C/G may be a protective factor against T2DM in the Han population^43^. The results of Chen et al.'s meta-analysis in 2015 showed that *SLC30A8* rs13266634 might be a crucial genetic contributor to the risk of T2DM among Asians and Europeans but not Africans. It also indicated that people with the CC genotype have a 33.0% and 16.5% higher risk of T2DM compared with those with TT and CT genotypes, respectively^14^. According to research by Zaahl et al., the promoter SNP rs7573065 (− 237 C/T) plays a protective role in the contribution of *SLC11A1* to inflammatory bowel disease^44^. They also revealed that when allele 3 of the 5′ microsatellite was present, the allele C to T alteration at the position of 237 (rs7573065) downregulated *SLC11A1* to a level comparable to that observed allele 2 of the microsatellite^45^.

Kissler et al. discovered that *SLC11A1* downregulation in NOD mice mimicked the protective Idd5.2 T1DM-resistant haplotype and decreased the prevalence of T1DM^46^. It was found that this gene affects the ability of dendritic cells (DCs) to process and present pancreatic islet antigens [i.e., glutamic acid decarboxylase GAD65], increasing the stimulation of a diabetogenic T-cell clone^47^. Unfortunately, there is a lack of evidence for the involvement of *SLC11A1* variants in the etiology of DM. In this regard, Yang and colleagues concluded that the *SLC11A1* gene variant rs3731685 (INT4) might be correlated with T1DM risk in a population of European ancestry. Although they found no correlation between mRNA levels of *SLC11A1* and different genotypes of this SNP in whole blood samples, a possible association with purified cell subsets, particularly monocytes or macrophages, could not be completely ruled out^48^. In another cohort study, Takahashi et al. examined the SNPs located in the promoter region of *SLC11A1*, which might affect transcriptional activity, in 224 controls and 95 Japanese cases of T1DM. Japanese participants have been found to carry the specified alleles 2, 3, and 7. They found a significant difference in the subset of Japanese individuals with T1DM; these patients had considerably higher allele 7 frequencies than the healthy subjects, as did those carrying no susceptibility HLA class II haplotypes, DR4-DQ4 or DR9-DQ9. Overall, they concluded that the new promoter variant of *SLC11A1* impacts Japanese individuals' susceptibility to T1DM^49^. *Mycobacterium avium subsp. paratuberculosis* (MAP) has been linked to the onset of T1DM; accordingly, Paccagnini et al. examined 59 T1DM cases and 79 healthy individuals for 9 *SLC11A1* SNPs and the presence of MAP using the PCR technique. Blood levels of MAP DNA and the 274C/T *SCL11A1* polymorphism were discovered to be linked to T1DM. Because MAP is not degraded by macrophages and is processed by DCs, it is important to determine whether mutant variants of *SLC11A1* affect the processing or presentation of MAP antigens, which could lead to an autoimmune disorder and T1DM^50^. In agreement with these reports, we found a negative association between T2DM and two SNPs in *SLC11A1* (rs3731864 G/A and rs17235416) and a positive association between the disease and *SLC11A1* rs3731865 G/C*.*

Both of the studied intronic variants were located in the splicing sites of the *SLC11A1* gene. Interestingly, results of our web-based analysis showed that the A allele of *SLC11A1* rs3731864 disrupts the binding sites of SC35, SF2/ASF, heterogeneous nuclear RNA protein (hnRNP) F, hnRNP H3, hnRNP H1, and hnRNP H2, whereas the minor allele of *SLC11A1* rs3731865 creates the binding site for SF2/ASF and hnRNP H3, as splicing factors. This is important because splicing factors are involved in regulating distinct gene expression processes^51^. It has been documented that alternative splicing via SF2/ASF^52^, hnRNP F^53,54^ can contribute to the pathogenesis of diabetes or cause insulin resistance. Furthermore, overexpression of the hnRNP H1 was also observed in the nucleus of Inflamed Islets of fulminant T1DM^55^. A distinct binding specificity has been reported between the human splicing factors ASF/SF2 and SC35, and these specificities are functionally important^56^.

An interaction network between proteins comprises a few highly connected nodes (known as hubs) and many poorly connected nodes. In genome-wide studies, it has been established that the deletion of a hub protein increases the likelihood of death, a phenomenon known as the centrality-lethality rule. A key notion of systems biology lies in the biological significance of network architectures, which are believed to reflect the special role hubs play in organizing networks^57^. Since proteins cannot act alone, most cellular functions depend on interactions between them. In the current study, we have utilized the inBio Discover™ to explore possible interactions between SLC11A1 and other proteins to gain valuable insight into a complex interaction network that may be responsible for the onset of T2DM. This is important since, to the best of our knowledge, the role of this solute carrier protein has not been studied in T2DM patients. Our Bioinformatics results showed that some of the genes that directly interact with SLC11A1 can also play important roles in the course of T2DM, making SLC11A1 a hub protein that can regulate different signaling pathways involved in the pathogenesis of T2DM. SLC11A1, a divalent cation transporter, plays an important role in early macrophage activation and exerts multiple pleiotropic effects on macrophage function, including on the expression of chemokines, IL-1β, tumor necrosis factor α (TNF-α)-inducible nitric oxide synthase, and MHC class II molecules. The multiple pleiotropic effects of SLC11A1 on macrophage function suggest that SLC11A1 is a prime candidate for T1DM in humans and mice^49^.

The main mediator of iron transfer is SLC11A2, and iron is absorbed through this apical transporter in intestinal epithelial cells and macrophages. Previous studies have demonstrated that iron metabolism can affect insulin sensitivity, leading to T2DM^58^. Ferroptosis is also associated with diabetic cognitive dysfunction, and a previous study has shown that Slc40a1 mediates ferroptosis in T1DM^59^. Accordingly, we found that SLC11A1 and ATPase copper transporting alpha (ATP7A) are the most relevant proteins interacting with SLC11A1. This is important since it has been established that ATP7A^60^, fibrinogen chain beta (FGB)^61^, fibrinogen chain alpha (FGA)^62^, Solute carrier family 11 member 2 (SLC11A2)^58^, and Solute carrier family 40 member 1 (SLC40A1)^63^ might have essential roles in the pathogenesis of T2DM. Interestingly, hemostatic dysfunction and subclinical inflammation might play a role in the complex etiopathogenesis of diabetic peripheral neuropathy (DPN). Fibrinogen is involved in both hemostatic and inflammatory pathways, and it is hypothesized that fibrinogen gene polymorphisms might be associated with DPN^64^. In general, various studies have shown this gene to be related to the pathogenesis of T2DM and SLC11A1. These evidences suggest that SLC11A1 may act as a regulatory hub for controlling cell’s metabolism and activity through controlling the activity of other genes. Further investigation on the relationship between these proteins and the investigated receptor are warranted.

Perversions from the normal pattern represent DNA distortion or base flipping in sequence^30^. From a clinical perspective, SNPs are potential diagnostic and therapeutic biomarkers for many types of cancer and metabolic disorders. Those in the promoter region affect gene expression by altering the promoter activity, binding of transcription factors, DNA methylation, and histone structure. Introns comprise approximately half of the human noncoding genome and have critical regulatory roles in gene regulation and expression. SNPs in intronic regions might cause diseases and alter the genotype–phenotype association by generating splice variants of transcripts and promoting or disrupting the binding and function of long noncoding RNAs (lncRNAs) (such as rs3731864 G/A and rs3731865 G/C). SNPs in the 5′-UTR regions can potentially affect translation, whereas those in the 3′-UTR region (i.e., rs17235416 + TGTG/−TGTG) impact the binding of microRNAs (miRNAs)^65,66^. Intronic sequences might be conserved, as they contain expression-regulating elements that impose functional constraints on their evolution^67,68^. SNPs located in these regions could be pathogenic even if they are conserved^69^. Variation analysis revealed that all three studied polymorphisms, specifically *SLC11A1* rs3731864 G/A and rs3731865 G/C, reside in unconserved regions across multiple mammalian species. Understanding the mechanisms underlying the effects of SNPs that result in metabolic diseases such as diabetes is critical for elucidating their molecular pathogenesis.

Based on the chromosomal position, rs3731864 is located 18 bp before exon 6, rs3731865 is located 14 bp after exon 4, and rs17235416 into exon 15 of the *SLC11A1* gene. The first and second variants are located in the regulatory regions; for example, splicing sites could potentially impact the expression of *SLC11A1*. Accordingly, the presence of a minor allele in these positions can affect post-transcriptional modifications and/or translation. Thus, it is hypothesized that nucleotide substitution in these locations is followed by producing a less or more efficient protein. However, the exact mechanism regarding the role of the SLC11A1-mutated protein is not understood yet and requires additional bioinformatics analyses.

Accordingly, rare functional noncoding SNPs identified by large-scale whole genome sequencing have revealed unexplained heritability of T2DM^70^ and can thus be considered valuable prognostic markers for the disease. This is crucial because the lack of prevention and healthcare measures accounts for the fast rise in the prevalence of such endocrine disorders and their complications in developing countries. Iran lacks the most recent studies and knowledge necessary for effectively managing and treating T2DM. Our research aims to develop effective and affordable approaches to assess the genetic variation-based risk of T2DM development. In order to provide better treatment options against T2DM, we expect that our findings may help clinicians in the management and early detection of this condition. Additionally, creating a T2DM biobank for this cohort will be beneficial since this is the first study testing these SNPs in T2DM patients. Although SNPs in the *SLC11A1* encoding gene's intronic and 3-UTR regions were chosen in the current study to study the relationship between *SLC11A1* variations and the risk of T2DM, these variants might not provide a full picture of the *SLC11A1* gene's genetic activity. As a result, a fine-mapping study may be needed subsequently. On the other hand, T2DM is a complicated metabolic disorder driven by environmental and genetic variables that were not examined in this study and can be considered a limitation. Furthermore, we have not performed Sanger sequencing to confirm our genotyping results, which can also be considered a limitation of the current study. Lastly, our sample size was relatively small, and this could potentially affect the outcome of such population-based studies. Despite these, we believe that the findings of our study highlight the essential role of *SLC11A1* polymorphisms in predisposition to T2DM in subjects with Iranian ancestry.

## Conclusion

Our findings showed that *SLC11A1* rs3731865 G/C is associated with an increased risk of T2DM in our population, while *SLC11A1* rs3731864 G/A and rs17235416 + TGTG/−TGTG SNPs were correlated to decreased risk of developing this endocrine disease. Further bioinformatics analyses, along with replicated studies on different ethnicities, are needed to confirm our findings. Additionally, given the significant effect of these SNPs on the onset of T2DM, it appears likely that additional genetic variants in this gene may contribute to T2DM susceptibility. These findings may facilitate a detailed understanding of the molecular pathogenesis of T2DM and the genetic basis of heterogeneous susceptibility, with potential implications for the development of more effective therapeutic strategies.

## Supplementary Information

Supplementary Information.

## Data Availability

All data relevant to the study are included in the article or uploaded as supplementary information. Furthermore, upon rational demand, the data will be accessible through the corresponding author.
