# Supplementary material for: Association of SLC11A1 polymorphisms with anthropometric and biochemical parameters describing Type 2 Diabetes Mellitus
Source: Sci Rep. 2023 Apr 16;13:6195. doi: 10.1038/s41598-023-33239-3 (PMC10106459; doi:10.1038/s41598-023-33239-3)
Supplement: Supplementary file 1 — Supplementary Information. [file 41598_2023_33239_MOESM1_ESM.docx]

**Supplementary Table S1.** Characteristics of primers used for genotyping studied *SLC11A1* gene polymorphisms.

| **Polymorphism** | **Function** | **Sequence** | **Genotyping Method** | **RE** | **Amplified Band (bp)** | **Annealing Temperature** |
| --- | --- | --- | --- | --- | --- | --- |
| rs3731864 G/A | Intronic Variant | F: GGCCAGATTCCTGTCTCCAGCC  R: TTCGATGTCAGAGCCCTTCT | PCR-RFLP | *MspI* | A: 236  G: 166 + 72 | 60**℃** |
| rs3731865 G/C | Intronic Variant | F: GACTTGGGATGCCCCATGTG  R: GGTACCACGAGCTCAGGGGC | PCR-RFLP | *ApaI* | G: 100 + 201  C: 301 | 61**℃** |
| rs17235416 +TGTG/-TGTG | 3′UTR | F (Del): TGACGTGACTGGCCTGCTGGTGA  F (Ins): TGACGTGACTGGCCTGCTGGTTG  FO: TCTCCCCAATTCATGGTTGCCC  RO: CACATGGCTGCGCTAGGAAAC | ARMS-PCR | *-* | Outer: 300  Ins: 124  Del: 120 | 59**℃** |

RE, restriction enzyme; bp, base pair; F, forward; R, reverse; FO, forward outer; RO, reverse outer; 3′-UTR, three prime untranslated regions; ins, insertion; del, deletion; PCR-RFLP, polymerase chain reaction-restriction fragment length polymorphism; ARMS-PCR, amplification refractory mutation system-polymerase chain reaction.

**Supplementary Table S2.** Haplotype analysis of *SLC11A1* gene polymorphisms on the risk of T2DM.

| **rs3731864 G/A** | **rs3731865 G/C** | **rs17235416 +TGTG/-TGTG** | **Case (%)** | **Control (%)** | **OR (95% CI)** | ***p*-value** |
| --- | --- | --- | --- | --- | --- | --- |
| G | C | +TGTG | 320 (80.0) | 306 (76.5) | 1 [reference] | |
| G | G | +TGTG | 47 (11.7) | 30 (7.5) | 1.50 (0.92-2.43) | 0.100 |
| A | C | +TGTG | 19 (4.8) | 38 (9.5) | 0.48 (0.27-0.85) | **0.010** |
| G | C | -TGTG | 10 (2.5) | 19 (4.75) | 0.50 (0.23-1.10) | 0.080 |
| A | G | +TGTG | 2 (0.5) | 4 (1.0) | 0.48 (0.09-2.63) | 0.386 |
| G | G | -TGTG | 1 (0.25) | 2 (0.5) | 0.48 (0.04-5.30) | 0.539 |
| A | C | -TGTG | 1 (0.25) | 1 (0.25) | 0.96 (0.06-15.36) | 0.975 |

T2DM, type 2 diabetes mellitus; ins, insertion; del, deletion; OR, odds ratio; CI, confidence intervals. Statistically significant interaction (*p* < 0.016) is shown in **Bold** and light gray.


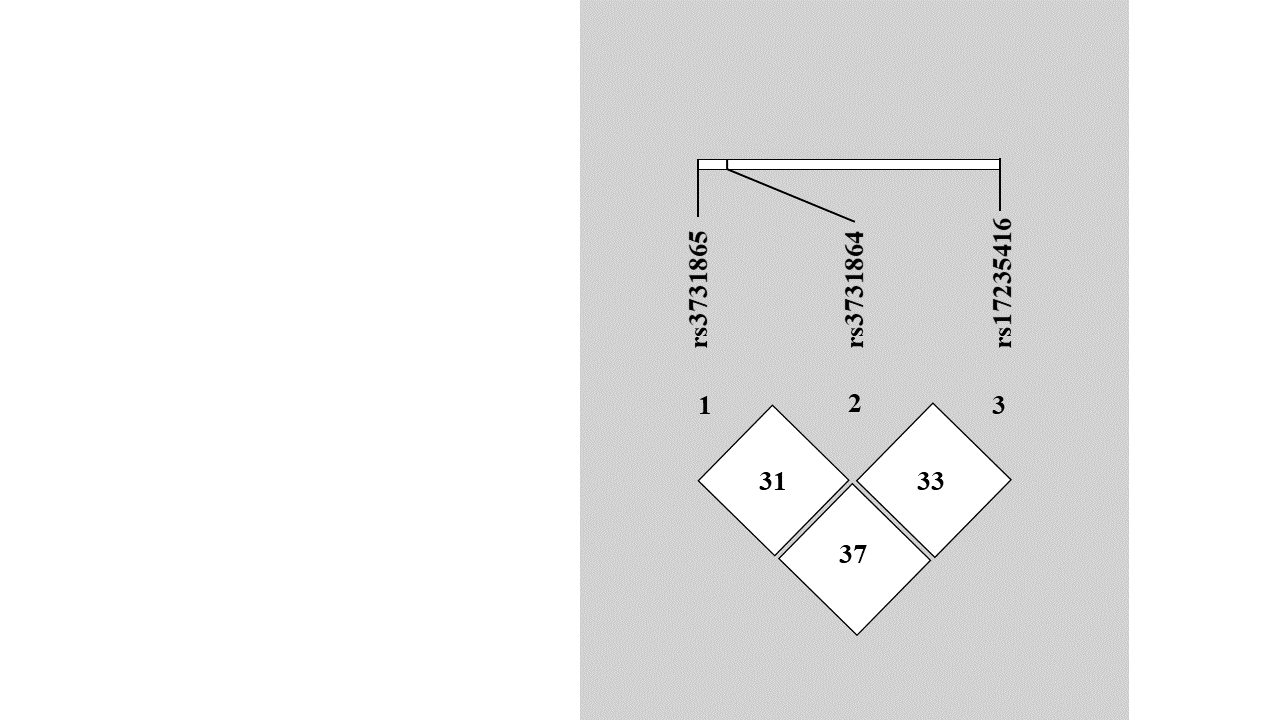


**Supplementary Figure S1.** Pairwise LD analysis of *SLC11A1*–rs3731864 G/A, –rs3731865 G/C, and –rs17235416 +TGTG/-TGTG polymorphisms.
